# Receptivity to malaria in the China–Myanmar border in Yingjiang County, Yunnan Province, China

**DOI:** 10.1186/s12936-017-2126-z

**Published:** 2017-11-21

**Authors:** Tianmu Chen, Shaosen Zhang, Shui-Sen Zhou, Xuezhong Wang, Chunhai Luo, Xucan Zeng, Xiangrui Guo, Zurui Lin, Hong Tu, Xiaodong Sun, Hongning Zhou

**Affiliations:** 1 Department of Malaria, National Institute of Parasitic Diseases, Chinese Center for Disease Control and Prevention, 207 Rui Jin Er Road, Shanghai, 200025 People’s Republic of China; 2 Key Laboratory of Parasite and Vector Biology, Ministry of Health, 207 Rui Jin Er Road, Shanghai, 200025 People’s Republic of China; 3 WHO Collaborating Centre for Tropic Diseases, 207 Rui Jin Er Road, Shanghai, 200025 People’s Republic of China; 4 National Center for International Research on Tropical Diseases, Ministry of Science and Technology, 207 Rui Jin Er Road, Shanghai, 200025 People’s Republic of China; 5 Yunnan Institute of Parasitic Diseases, Puer, People’s Republic of China; 6 Yingjiang County Center for Disease Control and Prevention, Dehong, People’s Republic of China

**Keywords:** Receptivity, Community structure, *Anopheles* community, Malaria transmission, Re-establishment, China–Myanmar border

## Abstract

**Background:**

The re-establishment of malaria has become an important public health issue in and out of China, and receptivity to this disease is key to its re-emergence. Yingjiang is one of the few counties with locally acquired malaria cases in the China–Myanmar border in China. This study aimed to understand receptivity to malaria in Yingjiang County, China, from June to October 2016.

**Methods:**

Light-traps were employed to capture the mosquitoes in 17 villages in eight towns which were categorized into four elevation levels: level 1, 0–599 m; level 2, 600–1199 m; level 3, 1200–1799 m; and level 4, > 1800 m. Species richness, diversity, dominance and evenness were used to picture the community structure. Similarity in species composition was compared between different elevation levels. Data of seasonal abundance of mosquitoes, human biting rate, density of light-trap-captured adult mosquitoes and larvae, parous rate, and height distribution (density) of *Anopheles minimus* and *Anopheles sinensis* were collected in two towns (Na Bang and Ping Yuan) each month from June to October, 2016.

**Results:**

Over the study period, 10,053 *Anopheles* mosquitoes were collected from the eight towns, and 15 *Anopheles* species were identified, the most-common of which were *An. sinensis* (75.4%), *Anopheles kunmingensis* (15.6%), and *An. minimus* (3.5%). *Anopheles minimus* was the major malaria vector in low-elevation areas (< 600 m, i.e., Na Bang town), and *An. sinensis* in medium-elevation areas (600–1200 m, i.e., Ping Yuan town). In Na Bang, the peak human-biting rate of *An. minimus* at the inner and outer sites of the village occurred in June and August 2016, with 5/bait/night and 15/bait/night, respectively. In Ping Yuan, the peak human-biting rate of *An. sinensis* was in August, with 9/bait/night at the inner site and 21/bait/night at the outer site. The two towns exhibited seasonal abundance with high density of the two adult vectors: The peak density of *An. minimus* was in June and that of *An. sinensis* was in August. Meanwhile, the peak larval density of *An. minimus* was in July, but that of *An. sinensis* decreased during the investigation season; the slightly acidic water suited the growth of these vectors. The parous rates of *An. sinensis* and *An. minimus* were 90.46 and 93.33%, respectively.

**Conclusions:**

The *Anopheles* community was spread across different elevation levels. Its structure was complex and stable during the entire epidemic season in low-elevation areas at the border. The high human-biting rates, adult and larval densities, and parous rates of the two *Anopheles* vectors reveal an exceedingly high receptivity to malaria in the China–Myanmar border in Yingjiang County.

## Background

Malaria remains a significant public health problem, especially in Africa and Southeast Asia. Owing to the inception of the World Health Organization (WHO)’s Mekong Malaria Programme a decade ago, the annual malaria incidence and mortality have declined continuously in the Greater Mekong Subregion (GMS) [1–3]. However, among the GMS nations, Myanmar has the heaviest disease burden of malaria and is one of the most threatening foci of malaria in Southeast Asia [4, 5]. The border of Kachin State in Myanmar has a high incidence and mortality rate of malaria [6]. It is thus crucial to assess the risk of malaria re-establishment in this border to allow the relevant departments in the region to develop optimal elimination strategies, since China and the GMS counties aim at malaria elimination by 2020 and 2030, respectively [7].

The infectivity-receptivity-vulnerability framework is an important method to assess the risk of malaria re-establishment in many countries [8–12]. The framework defines receptivity as the presence, distribution, seasonal abundance and bionomics of the potential vector [8, 9, 12]. Control of malaria transitions depends on integrated actions [7], and according to “A framework for malaria elimination” announced by the WHO, receptivity is a key point to malaria re-emergence [13]. Considering the high cost of measuring receptivity in an area, it is systematically difficult to obtain first-hand data on receptivity in the China–Myanmar border.

In China, malaria is being rapidly eliminated [7, 14, 15], which has been mainly attributed to malaria control in the China–Myanmar border in Yunnan Province. In 2014 and 2015, Yingjiang County was one of the few counties to report malaria transmission. In 2016, it was the only county to report locally acquired malaria cases in the border. Therefore, it is specifically important to determine receptivity to malaria in this county.

In Southeast Asia, including China and Myanmar, deforestation and cultivation of cash crops (such as banana, rubber, and maize) constitute the most important environmental changes in rural areas [16–18]. For example, field investigation and interview of the local primary public health care provider revealed that the main crop of these regions was rice, which occupied approximately 2 million square kilometres in Na Bang town, Yingjiang County, before 2005. After 2005, banana was grown as the main crop in these regions. Until early 2016, the area of banana production had increased to > 3 million square kilometres, and no rice crops were left. These changes may have led to alterations in the population density, life history [19], and behaviour of vectors such as laying eggs [20]. This change in ecotope in the China–Myanmar border may have resulted to change in receptivity to malaria in the region.

The main malaria vectors in the China–Myanmar border are *Anopheles minimus* and *Anopheles sinensis* [5, 19, 21, 22]; the major vectors in China are *An. sinensis*, *Anopheles lesteri*, *Anopheles dirus*, and *An. minimus* [23, 24]. In recent years, *An. minimus* in these areas has become the focus of research. Several studies investigated the ecological features of malaria vectors, including species composition and population dynamics, density, human blood index, proportion of sporozoites, and environmental factors (e.g., land use and land cover changes) [6, 18, 19, 21]. These studies provided valuable information for generating targeted intervention strategies for malaria control and elimination along the border areas. However, the community structure of *Anopheles* mosquitoes at different elevations in the border remains unknown. Moreover, the seasonal receptivity in the county, especially the seasonality of the human-biting rate and larval density, has not been well investigated in recent years.

Therefore, this study aimed to collect mosquitoes from 17 villages in eight towns in Yingjiang County at different elevations; analyse the community structure by species richness, diversity, dominance, and evenness [6, 25–32]; and examine receptivity to major malaria vectors (*An. minimus* and *An. sinensis*) in the China–Myanmar border.

## Methods

### Study area

Yingjiang County (24°24ʹ to 25°20ʹN, 97°31ʹ to 98°16ʹE), located in the west of Yunnan Province, has a population of > 0.3 million, includes 15 towns and 103 villages, and has a boundary line of 214.6 km. Its climate is warm and humid at low altitudes and cold at high altitudes. The main cash crops are rice, banana, coffee, sugarcane, and maize. Buffalo, yellow cattle, pigs, and dogs are also common. This variety in climate, ecology, and environment makes the county favourable for malaria vectors.

Two towns (Na Bang and Ping Yuan) in the county were selected as sentinel sites from June to October 2016. Na Bang, bordering on Myanmar and located west of the county, has a boundary line of 20.5 km and has nine villages, with a total population of 1751. The main cash crop is banana. The town has a tropical-subtropical climate and a low elevation, with the lowest elevation of 210 m. The average annual temperature is 22.7 °C, and the average annual precipitation is 2655 mm. Ping Yuan, the capital town of the county, has a population of 53.5 thousand and has 85 villages. Its main cash crop is rice, and its elevation is 937 m.

**Fig. 1:**
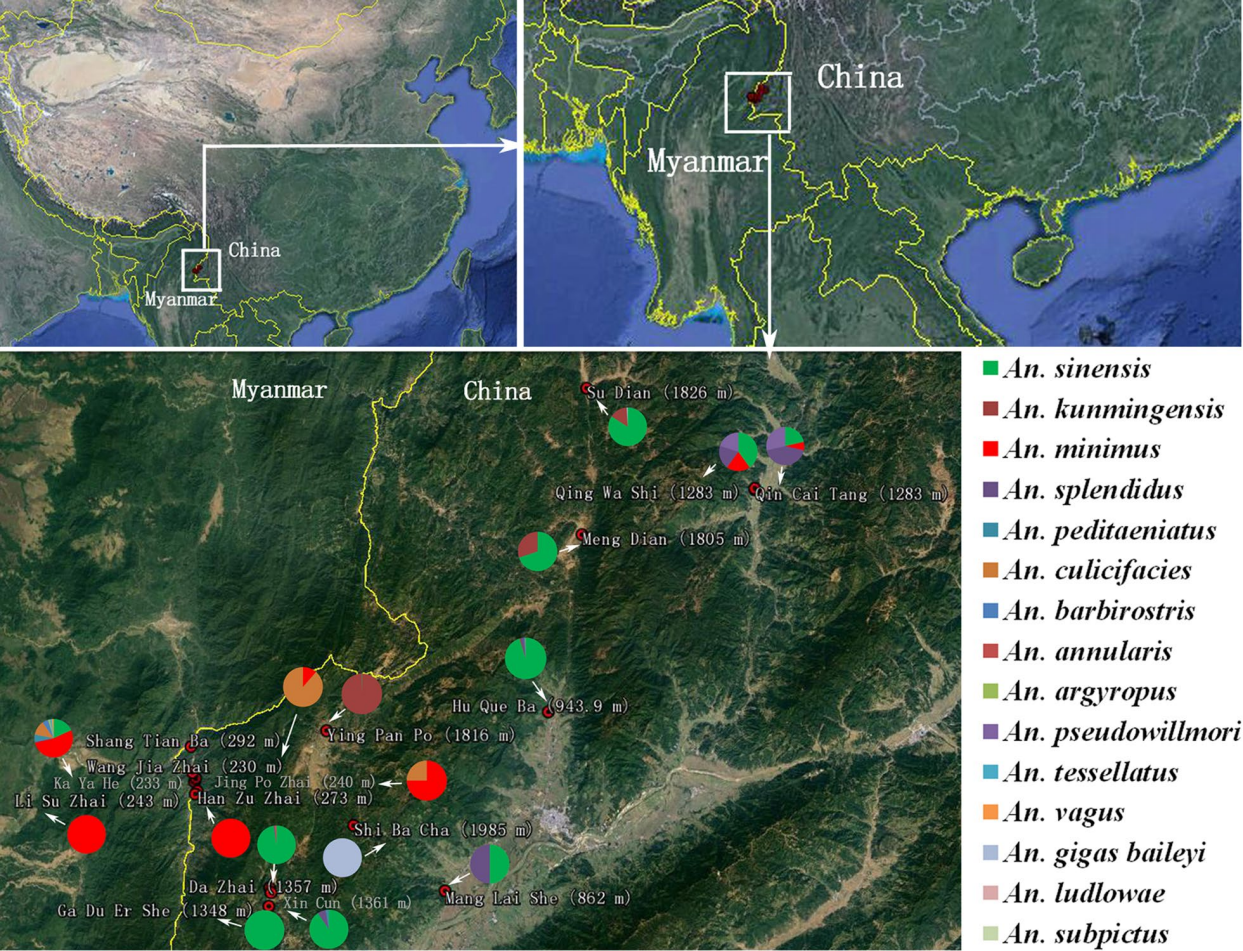
Locations of study sites and a pie-chart showing *Anopheles* distribution (percentage) in Yingjiang County. (No *Anopheles* mosquito was captured in Shang Tian Ba.)

In this study, 17 villages in eight towns were included and categorized into four levels according to the elevation of the study sites (Fig. 1): level 1, 0–599 m; level 2, 600–1199 m; level 3, 1200–1799 m; and level 4, > 1800 m (Table 1). In May and October 2016, a cross-sectional study was conducted on the community structure of *Anopheles* mosquitoes in the 17 villages. To determine the seasonal abundance of mosquitoes, the human-biting rate (*ma*) [19], density of light-trap-captured adult mosquitoes and larvae, parous rate [20], and height distribution (density) of *An. minimus* and *An. sinensis* were investigated in Na Bang and Ping Yuan each month from June to October 2016 (Table 2).

**Table 1:** Global positioning system information of selected survey sites at different elevations

Elevation levels	Town	Village	Latitude	Longitude
1 (0 m ~)	Na Bang	Ka Ya He	24.72171	97.569687
		Jing Po Zhai	24.724932	97.570903
		Wang Jia Zhai	24.7302	97.567
		Han Zu Zhai	24.713553	97.573415
		Li Su Zhai	24.710657	97.571707
		Shang Tian Ba	24.753889	97.563333
2 (600 m ~)	Ping Yuan	Hu Que Ba	24.809265	97.924073
	Tai Ping	Mang Lai She	24.639444	97.832944
3 (1200 m ~)	Zhi Na	Qing Wa Shi	25.02815	98.117844
		Qin Cai Tang	25.026906	98.118117
	Tong Bi Guan	Xin Cun	24.631647	97.657962
		Ga Du Er She	24.614827	97.65857
		Da Zhai	24.627342	97.659831
4 (1800 m ~)	Tai Ping	Shi Ba Cha	24.694127	97.738692
	Meng Nong	Meng Dian	24.97327	97.945571
	Su Dian	Su Dian	25.108227	97.939872
	Xi Ma	Ying Pan Po	24.777837	97.703559

**Table 2:** Height distribution of malaria vectors in selected towns in Yingjiang County

Elevation levels	Town	Number of villages	Number of light-trap nights
1 (0 m ~)	Na Bang	6	92
2 (600 m ~)	Ping Yuan	1	77
	Tai Ping	1	4
3 (1200 m ~)	Zhi Na	2	5
	Tong Bi Guan	3	4
4 (1800 m ~)	Tai Ping	1	3
	Meng Nong	1	2
	Su Dian	1	2
	Xi Ma	1	2
Total		17	191

### Mosquito collection and species identification

Centers for Disease Control and Prevention (CDC) light-traps without bait were used to capture mosquitoes. After transport to the laboratory, the mosquitoes were morphologically separated as *Anopheles*, *Culex*, *Aedes*, and other subfamilies or genera [6]. *Anopheles* mosquitoes were further morphologically sorted according to their species [6, 21]. After identifying the samples, each mosquito was kept in a cryogenic vial (Corning Inc., NY, USA) using 75% alcohol and stored in a − 20 °C freezer immediately to prepare for DNA extraction and identification using multiplex polymerase chain reaction (PCR). The DNA of *An. minimus* groups and the Hyrcanus Group were extracted from legs or wings of each mosquito for further species confirmation [33, 34].

### Human-biting rate

In each sentinel town, two survey sites (inner and outer) in each village were used for surveillance of the human-biting rate of *An. minimus* and *An. sinensis*. Although the human-landing catch (HLC) is the gold standard for monitoring mosquitoes that bite humans [35–37], it is labour intensive, cumbersome, and hazardous and requires intense supervision [38]. Alternatively, the human-baited double-net (HDN) trap is a simple and cheap method to estimate the human-biting rate outdoors without exposing collectors to vector bites [39]; and so far the best-performing trap, with similar efficiency to HLC. In this study, the human-biting rate of *An. minimus* and *An. sinensis* was monitored for 10 nights in four houses using the HDN trap from June to October 2016. One local volunteer was employed to rest inside a small bed net and was consequently fully protected from mosquitoes for the whole night’s duration. A larger bed net was hung over the smaller net and raised 30 cm above the ground. Both nets were protected from the elements by plastic-sheeting roof, but were not treated with any insecticide. One specialized person captured the mosquitoes from inside and outside the larger bed net per hour from 2000 to 0700 h. The species were then identified in the laboratory, and the number of captured mosquitoes was recorded at each survey site to calculate the human-biting rate.

### Seasonal abundance of adult mosquitoes

The vectors in human bedrooms and cattle shelters were captured using CDC light-traps from June to October 2016, and the seasonal abundance in terms of density (per light-trap per night) of *An. minimus* and *An. sinensis* was calculated accordingly. In each village, four light-traps were hung separately in two human bedrooms and two cattle shelters per night, from 2000 to 0700 h of the next day. In each month, vectors were captured twice on two nights at the same place.

### Density of larvae

All kinds of breeding sites (bogs, slow-flowing water bodies, rice paddies, pools, and ditches) of the two vectors were surveyed in the two towns each month during the survey season. Standard dippers with approximately 500 mL volume were used to collect larvae from the water bodies [40]. Ten dips of water were taken to determine the presence of anophelines. If anophelines were present, the larvae in the 10 dips were collected in a small bottle with some water. The bottles were then numbered and transported to the laboratory to count the number of first-, second-, third-, and fourth-instar larvae and pupae of the two vectors [41]. The species of late third- and fourth-instar anopheline larvae were identified under a microscope using commonly accepted guidelines [42]. The identified larvae were preserved in a cryogenic vial (Corning Inc.) containing 75% alcohol for further identification by PCR. The density of larvae (per 10 dips) was calculated accordingly. Additionally, the pH value and location of the breeding sites were surveyed to analyse the relationship between these factors and the density of larvae.

### Parous rate

Landing collections were performed by collecting *An. minimus* and *An. sinensis* in cattle shelters in Na Bang and Ping Yuan each month, from 2130 to 2200 h per night. Mosquitoes were collected by four collectors using an aspirator. The collected mosquitoes were transported to the laboratory of Yingjiang CDC, where they were killed using chloroform and dissected using minute dissection needles to collect their ovaries. The ovaries were separated from the other internal organs (including the Malpighian tubules and stomach) and teased apart at approximately 40× magnification through a dissection microscope to confirm whether the mosquitoes had laid eggs. The parous rate was calculated accordingly.

### Height distribution

The height of each site in the 17 villages of the eight towns, where a light-trap was hung, was recorded using a handset global positioning system (Garmin International Inc., Olathe, KS, USA) to analyse the relationship between the height and the density of the two vectors. The lowest elevation was 210 m in Na Bang town, and the highest was approximately 2000 m in Shi Ba Cha village in Tai Ping town. As mentioned, the towns were divided into four levels. Besides the two sentinel towns, six towns were selected at different elevation levels. The same mosquito-capturing method was adopted as the one used for investigating the seasonal abundance of adult mosquitoes. Thereafter, the captured mosquitoes were transported to the laboratory to confirm whether they were the target vectors.

### Data analysis

Species richness of *Anopheles* mosquitoes was measured using the index *N,* which represents the number of species [6]. Species dominance was measured by the Berger–Parker dominance index *d,* which was equal to the fraction of a species with a majority proportion in the study site or area [6]. Species diversity and evenness were evaluated by three indices—Simpson diversity index *D*, Shannon diversity index *H*, and evenness index *E* [25–32]. Similarity among different elevation levels was measured using the Morisita–Horn similarity index *C* [32–43]. The indices *D* and *H* were calculated from the proportion of each species; *E,* also known as Shannon’s equitability, was calculated by dividing *H* by richness; and *C* was calculated by the number of individuals of each species and the total number of mosquitoes [43]. These indices were represented by the following equations:

$$D = 1 - \mathop \sum \limits_{n = 1}^{N} p_{i}^{2}$$

$$H = - \mathop \sum \limits_{n = 1}^{N} p_{i} \ln p_{i}$$

$$E = \frac{H}{\ln N}$$

$$C = \frac{{2\mathop \sum \nolimits n_{1i} n_{2i} }}{{\left( {\lambda_{1} + \lambda_{2} } \right)M_{1} M_{2} }}, \quad \lambda_{i} = \frac{{\mathop \sum \nolimits n_{ji}^{2} }}{{M_{j}^{2} }}$$

where *N* is the richness index, *p*_*i*_ is the proportion of a species that belongs to the *i*th species, *n*_*ji*_ is the number of individuals of a species *i* in an area *j*, and *M*_*j*_ is the number of individuals in an area *j*.

In the cross-sectional study, the light-trap density (females/trap/night), *N*, *D*, *H*, *d*, and *E* were evaluated to determine the community structure of *Anopheles* mosquitoes in the 17 villages, and *C* was used to measure the similarity among different elevation areas. The community-structure indicators were used to examine the population dynamics at the two surveillance sites.

Microsoft Excel 2010 (Microsoft Corp., USA) was employed to represent the data. Data analysis was performed using SPSS 13.0 software. Differences between larvae and pH value of water were calculated using the Pearson correlation test and Chi square test. Differences between population density and height were calculated using the Pearson correlation test and curve fitting of the statistical model with observed data.

## Results

### Community structure and population dynamics of *Anopheles* mosquitoes

Over the study period, 191 trap nights were conducted, and 56,834 mosquitoes were collected in 17 villages. The majority of captured mosquitoes were *Culex* (45,180, 79.5%), followed by *Anopheles* (10,053, 17.7%), *Aedes* (1430, 2.5%), and other subfamilies or genera (171, 0.3%). Fifteen *Anopheles* species were identified and observed in the samples: *An. sinensis* (75.4%), *Anopheles kunmingensis* (15.6%), and *An. minimus* (3.5%), followed by 12 other *Anopheles* species (5.5%) (Table 3). The *Anopheles* distribution in each village is shown in Fig. 1.

**Table 3:** *Anopheles* species composition by elevation and pooled across study sites and study period

Species	Composition by elevation				Pooled	
	0 m ~	600 m ~	1200 m ~	1800 m ~	n	%
*An. sinensis*	16.91	95.29	91.99	17.96	7579	75.39
*An. kunmingensis*	0.00	0.00	0.00	81.58	1572	15.64
*An. minimus*	51.20	0.36	0.89	0.00	351	3.49
*An. splendidus*	0.64	3.07	4.09	0.00	240	2.39
*An. culicifacies*	16.43	0.01	0.00	0.00	104	1.03
*An. peditaeniatus*	5.74	0.91	0.71	0.05	104	1.03
*An. barbirostris*	4.63	0.01	0.00	0.00	30	0.30
*An. annularis*	0.00	0.19	0.89	0.00	18	0.18
*An. argyropus*	1.44	0.07	0.00	0.00	14	0.14
*An. pseudowillmori*	0.00	0.06	1.42	0.00	12	0.12
*An. tessellatus*	1.59	0.00	0.00	0.00	10	0.10
*An. vagus*	1.44	0.00	0.00	0.00	9	0.09
*An. gigas baileyi*	0.00	0.00	0.00	0.42	8	0.08
*An. ludlowae*	0.00	0.01	0.00	0.00	1	0.01
*An. subpictus*	0.00	0.01	0.00	0.00	1	0.01

The area with a level 1 elevation had the lowest *Anopheles* density (6.82 females/trap/night) and dominance index (*d* = 0.51), but the highest Simpson diversity index (*D* = 0.68), Shannon diversity index (*H* = 1.47), and evenness index (*E* = 0.67) (Table 4). Furthermore, the richness index (*N* = 9) in such area was lower than that of a level 2 area, but higher than those of level 3 and 4 areas. A level 2 area had the highest species richness index (*N* = 11) and dominance index (*d* = 0.95), but the lowest diversity indices *D* (0.09) and *H* (0.24) and evenness index *E* (0.10). Compared with a level 1 area, level 3 and 4 areas had lower *N*, *D*, *H* and *E* indices but higher *d* index. Among all *Anopheles* species, *An. minimus*, *An. sinensis*, and *An. kunmingensis* showed the highest proportion in areas of elevation levels 1, 2/3, and 4, respectively.

**Table 4:** Population density and community structure of *Anopheles* mosquitoes at each elevation level

Elevation (m)	Density (f/t/n)	*N*	Diversity index		*d*	*E*
			*D*	*H*		
0 ~	6.82	9	0.68	1.47	0.51	0.67
600 ~	85.64	11	0.09	0.24	0.95	0.10
1200 ~	62.44	6	0.15	0.39	0.92	0.22
1800 ~	214.11	4	0.30	0.50	0.82	0.36

f/t/n, females/trap/night; *N*, species richness; *D*, Simpson diversity index; *H*, Shannon diversity index; *d*, dominance index; *E*, evenness index

**Fig. 2:**
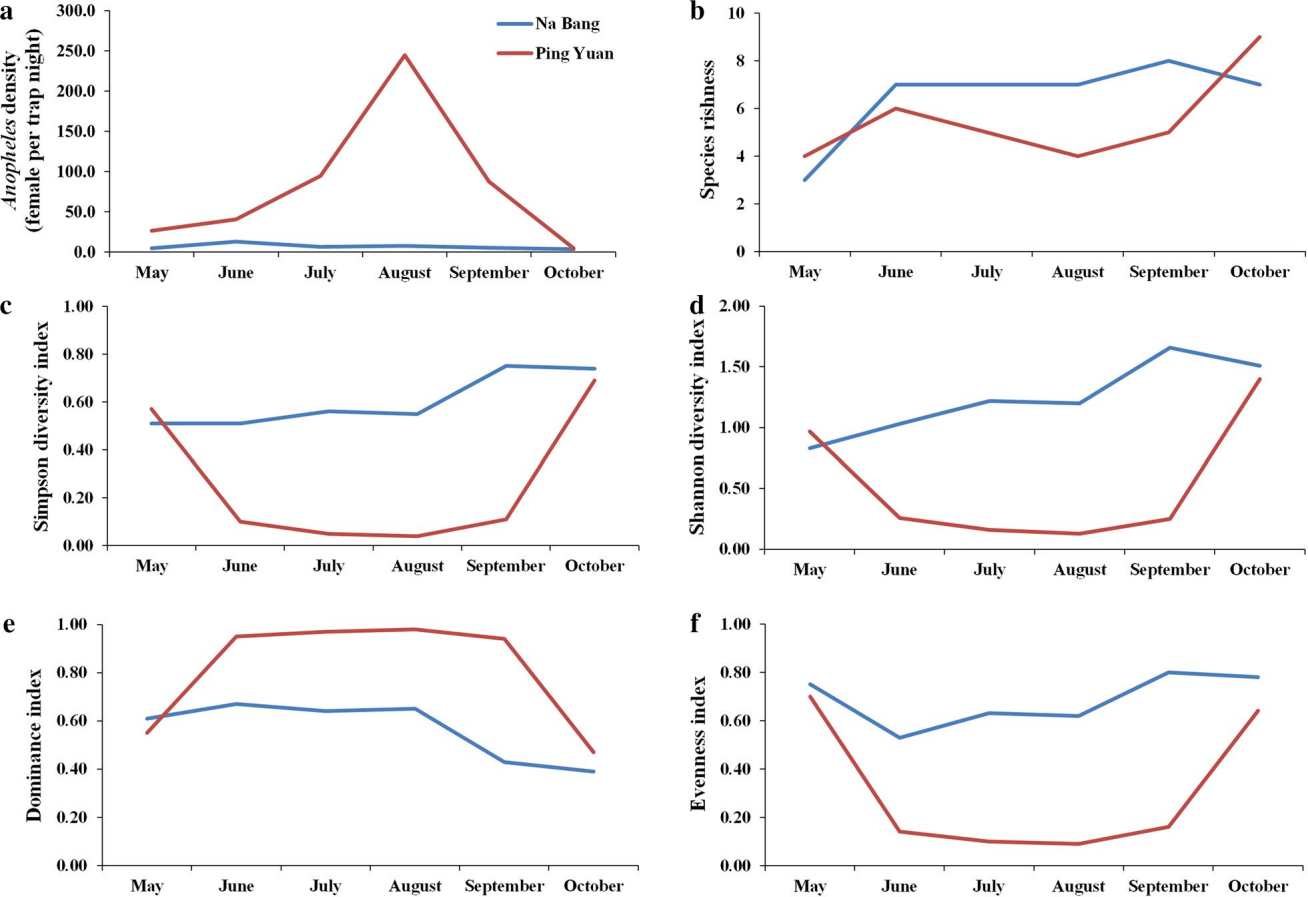
Population dynamics of *Anopheles* mosquitoes at two study sites in Yingjiang County, May–October 2016. **a** Pooled population density (females/trap/night) of all *Anopheles* species. **b** Species richness. **c** Simpson diversity index. **d** Shannon diversity index. **e** Dominance index. **f** Evenness index

Similarity analysis showed that the species composition of level 2 and 3 areas had the highest similarity (Morisita–Horn index *C* = 0.999), but any other two level areas showed low similarities (Morisita–Horn index *C* range, 0.059–0.274) (Table 5). The results of the two surveillance sites showed that the major *Anopheles* species in Na Bang was *An. minimus*, followed by *An. sinensis* (Table 6). In Na Bang, the pooled density of the entire study season was 6.82 females/trap/night, with a peak of 13.06 females/trap/night in June. Moreover, Na Bang had the highest *N* (8), *D* (0.75), *H* (1.66), and *E* (0.8) indices in September, but the highest *d* (0.67) index in June. All indicators of community structure in Na Bang showed a low variation during the season. In contrast, a large variation in these parameters was observed in Ping Yuan. The pooled density of the entire study season was 89.99 females/trap/night, with a peak of 244.60 females/trap/night in August. Additionally, the highest *N* (9), *D* (0.69), and *H* (1.46) indices were observed in October, but the highest *d* (0.98) index was observed in August, and the highest *E* (0.70) index in May (Fig. 2).

**Table 5:** Similarity in species composition between different elevations

	0 m ~	600 m ~	1200 m ~	1800 m ~
0 m ~	1			
600 m ~	0.266	1		
1200 m ~	0.274	0.999	1	
1800 m ~	0.059	0.213	0.214	1

**Table 6:** *Anopheles* species composition by month

Town	Species	May	June	July	August	September	October	Pooled	
								n	%
NB	*An. minimus*	33.33	66.99	63.89	65.25	8.54	17.86	321	51.20
	*An. sinensis*	5.56	19.62	10.19	5.93	42.68	16.07	106	16.91
	*An. culicifacies*	61.11	6.70	12.04	12.71	7.32	39.29	103	16.43
	*An. peditaeniatus*	0.00	1.91	0.00	7.63	13.41	21.43	36	5.74
	*An. barbirostris*	0.00	2.87	6.48	1.69	15.85	1.79	29	4.63
	*An. tessellatus*	0.00	1.44	1.85	3.39	1.22	0.00	10	1.59
	*An. vagus*	0.00	0.00	3.70	3.39	1.22	0.00	9	1.44
	*An. argyropus*	0.00	0.00	0.00	0.00	9.76	1.79	9	1.44
	*An. splendidus*	0.00	0.48	1.85	0.00	0.00	1.79	4	0.64
PY	*An. sinensis*	54.55	94.60	97.23	97.75	94.37	45.95	6606	95.34
	*An. splendidus*	36.36	4.01	1.19	1.10	4.35	29.73	209	3.02
	*An. peditaeniatus*	0.00	0.00	0.00	1.02	1.07	10.81	63	0.91
	*An. minimus*	6.82	0.77	0.13	0.13	0.14	4.05	25	0.36
	*An. annularis*	2.27	0.15	1.19	0.00	0.00	0.00	13	0.19
	*An. argyropus*	0.00	0.00	0.26	0.00	0.07	2.70	5	0.07
	*An. pseudowillmori*	0.00	0.31	0.00	0.00	0.00	2.70	4	0.06
	*An. barbirostris*	0.00	0.00	0.00	0.00	0.00	1.35	1	0.01
	*An. ludlowae*	0.00	0.00	0.00	0.00	0.00	1.35	1	0.01
	*An. culicifacies*	0.00	0.00	0.00	0.00	0.00	1.35	1	0.01
	*An. subpictus*	0.00	0.15	0.00	0.00	0.00	0.00	1	0.01

*NB* Na Bang, *PY* Ping Yuan

### Human-biting rate

**Fig. 3:**
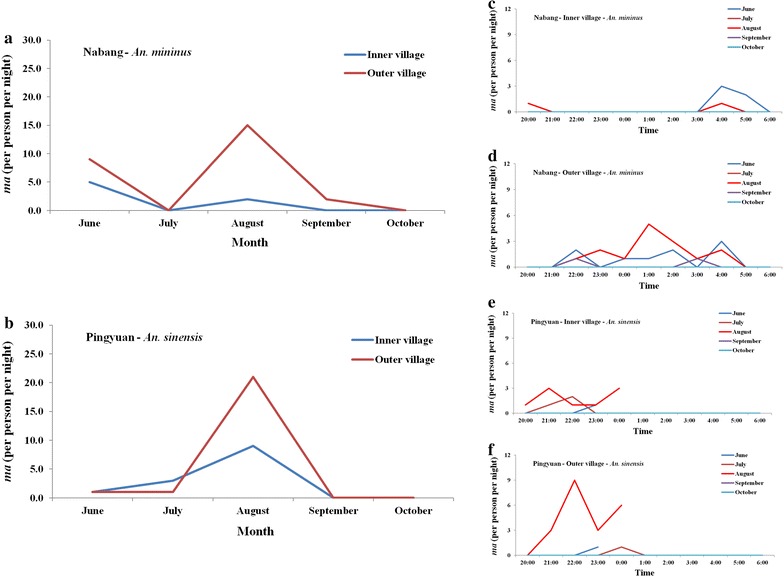
Seasonality of human-biting rate of *Anopheles minimus* and *Anopheles sinensis* in two surveillance sites. **a** Human-biting rate of *An. minimus* at the inner and outer village sites of Na Bang from June to October 2016. **b** Human-biting rate of *An. sinensis* at the inner and outer village sites of Ping Yuan from June to October 2016. **c** Human-biting rate (per hour) of *An. minimus* at the inner village site of Na Bang. **d** Human-biting rate (per hour) of *An. minimus* at the outer village site of Na Bang. **e** Human-biting rate (per hour) of *An. sinensis* at the inner village site of Ping Yuan. **f** Human-biting rate (per hour) of *An. sinensis* at the outer village site of Ping Yuan. Data on the human-biting rate in Ping Yuan were only collected at 23:00 h in June and 24:00 h in August because of intense rainfall on those nights

The human-biting rate of *An. minimus* was 1.4/bait/night at the inner survey site, but 5.2/bait/night at the outer survey site in Na Bang from June to October 2016. Meanwhile, the human-biting rate of *An. sinensis* was 0/bait/night in Na Bang, irrespective of the location. At the inner site, the peak human-biting rate of *An. minimus* was in June, with 5/bait/night (Table 7). However, at the outer site, although the human-biting rate of *An. minimus* was 9/bait/night in June, the peak was 15/bait/night (Fig. 3). *Anopheles minimus* was more likely to attack at 0100 and 0400 h at the inner site, but only at 0400 h at the outer site (Fig. 3).

**Table 7:** Human-biting rate of *An. minimus* and *An. sinensis* in Na Bang and Ping Yuan from June to October 2016

Towns	Location	Number of bait	Number of night	Number of *An. minimus*	Number of *An. sinensis*	*ma* of *An. minimus* (per bait per night)	*ma* of *An. sinensis* (per bait per night)
Na Bang	Inner village	1	5	7	0	1.4	0
	Outer village	1	5	26	0	5.2	0
Ping Yuan	Inner village	1	5	0	13	0	2.6
	Outer village	1	5	0	23	0	4.6

In Ping Yuan, the human-biting rate of *An. minimus* was 0/bait/night, irrespective of the location. However, the human-biting rate of *An. sinensis* was 2.6/bait/night at the inner site and 4.6/bait/night at the outer site. The peak human-biting rate of *An. sinensis* was in August, with 9/bait/night at the inner site and 21/bait/night at the outer site (Fig. 3). *Anopheles sinensis* was more likely to attack at 2100 and 2400 h at the inner site and 2200 and 2400 h at the outer site (Fig. 3).

### Seasonal abundance of adult mosquitoes

In Na Bang, the major vector was *An. minimus*. Its peak density was observed in human bedrooms in May (5 females/trap/night) and in cattle shelter in June (13.25 females/trap/night). There were two peaks (June and September) of *An. sinensis* in cattle shelters in the town, with densities of 4.5 females/trap/night and 4.25 females/trap/night, respectively. However, in human bedrooms, the density of *An. sinensis* decreased from May to October.

**Fig. 4:**
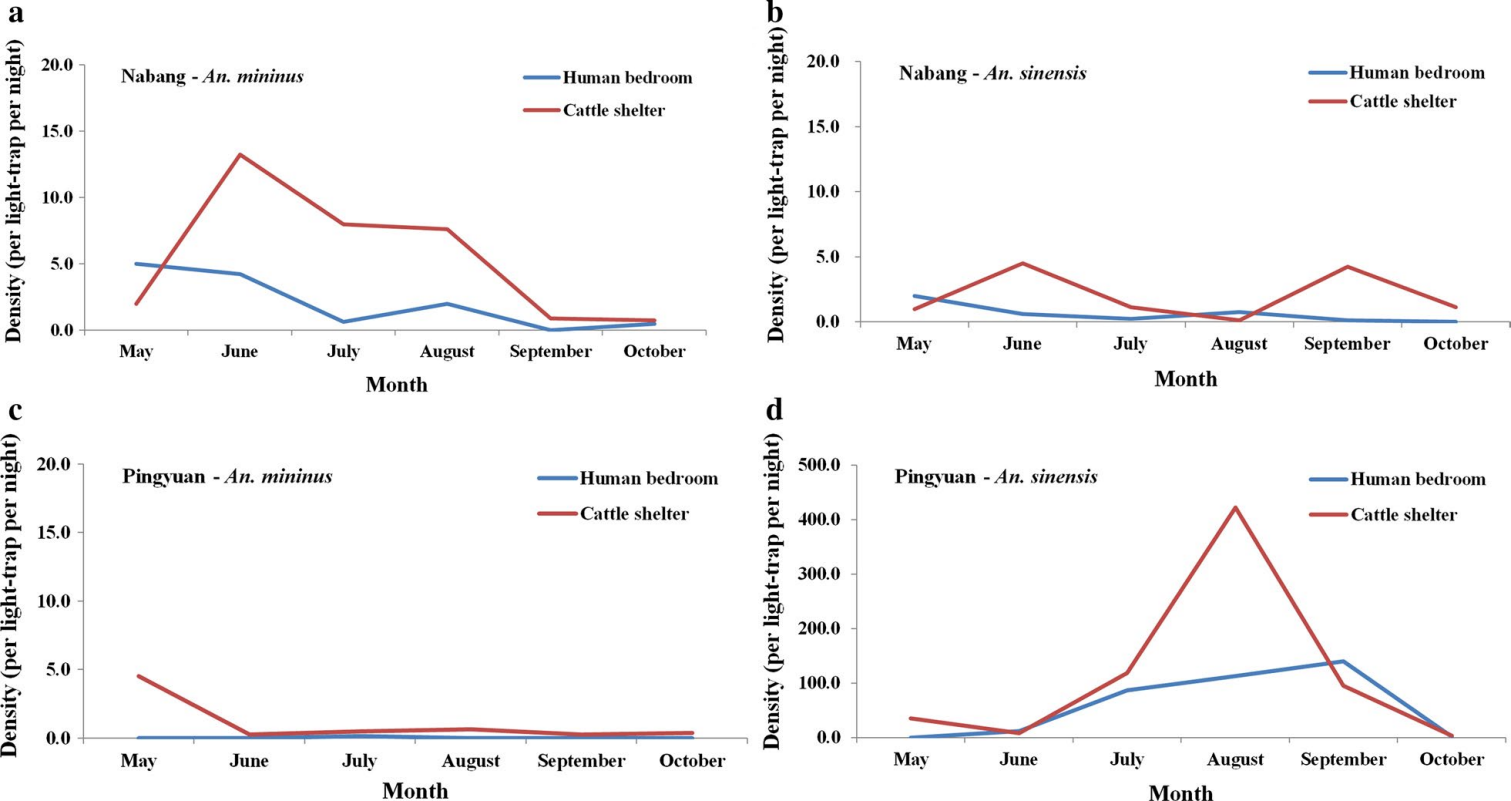
Seasonality of trap-captured *Anopheles minimus* and *Anopheles sinensis* at the two surveillance sites. **a** Density of *An. minimus* in human bedrooms and cattle shelters in Na Bang. **b** Density of *An. sinensis* in human bedrooms and cattle shelters in Na Bang. **c** Density of *An. minimus* in human bedrooms and cattle shelters in Ping Yuan. **d** Density of *An. sinensis* in human bedrooms and cattle shelters in Ping Yuan

In Ping Yuan, the major vector was *An. sinensis*, the peak density of which was found in cattle shelters in August (422 females/trap/night) and in human bedrooms in September (140.25 females/trap/night). However, the density of *An. minimus* was low, and the seasonality of this vector was not evident in the town (Fig. 4).

### Density of larvae

**Fig. 5:**
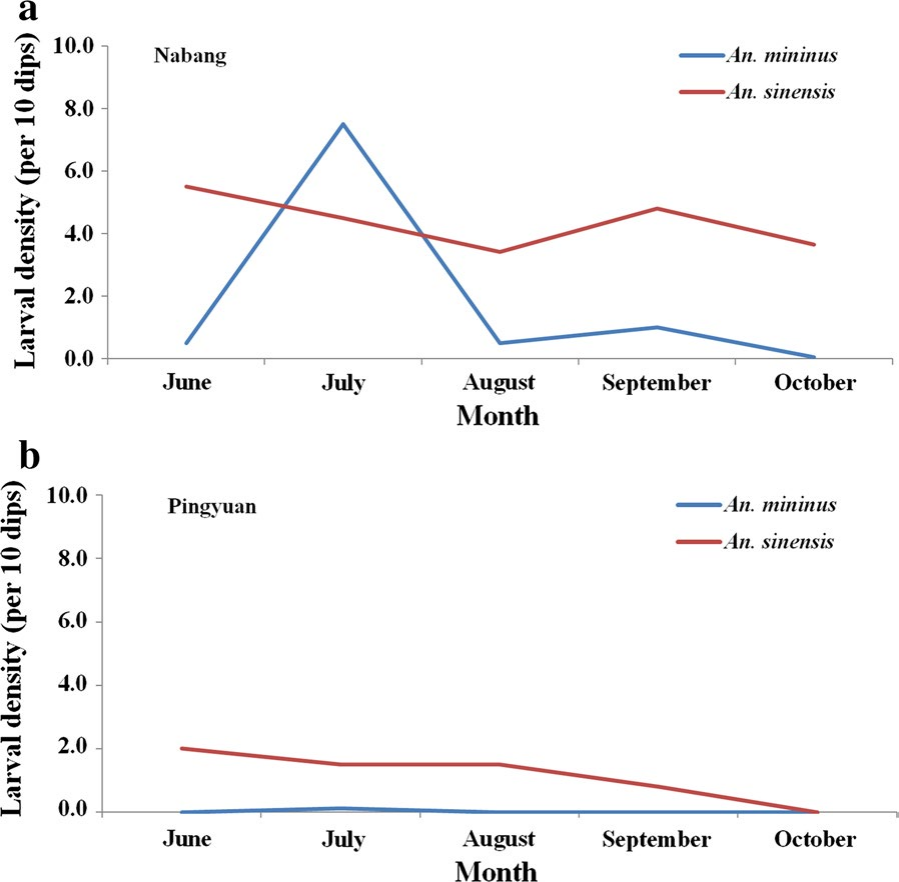
Seasonal abundance of larval density of *Anopheles minimus* and *Anopheles sinensis* at two surveillance sites. **a** Larval density in Na Bang. **b** Larval density in Ping Yuan

The seasonality of *An. minimus* larvae was evident in Na Bang, with a density of 7.5/10 dips. Meanwhile, the peaks of *An. sinensis*—in Na Bang were in June and September, with densities of 5.5/10 dips and 4.8/10 dips, respectively. Ping Yuan had a lower density of both vectors, and the density of *An. sinensis* decreased during the investigation season. No larva of *An. minimus* was detected in Ping Yuan during the investigation (Fig. 5).

**Fig. 6:**
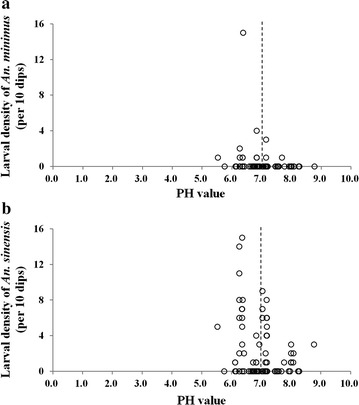
pH value and larval density of *Anopheles minimus* and *Anopheles sinensis* in Yingjiang County. **a** *An. minimus.* **b** *An. sinensis*

A total of 87 samples were collected from different water bodies. There was a significant difference between the larvae of *An. minimus* and pH of the water samples surveyed (*χ* ^2^ = 4.721, *P* = 0.030; Table 8). In contrast, no significant difference existed between the larvae of *An. sinensis* and pH value of the water samples surveyed (*χ* ^2^ = 0.001, *P* = 0.976). However, the density of the larvae was negatively correlated with the pH value; the correlation coefficient (*r* = − 0.297, *P* = 0.005) was calculated by the Pearson correlation test. Water samples with low pH showed a high density of *An. sinensis* larvae (Fig. 6).

**Table 8:** Relationship between pH value and larvae of the two vectors

	pH < 7.0	pH > 7.0	Total
*An. minimus*	40	47	87
Positive	9	3	12
Negative	31	44	75
*An. sinensis*	40	47	87
Positive	20	26	46
Negative	20	21	41

### Parous rate

In this study, 283 *An. sinensis* mosquitoes captured in cattle shelters were dissected, among which 256 (90.46; 95% confidence interval, 87.04–93.88%) had laid eggs. Fifteen *An. minimus* mosquitoes captured in cattle shelters were also dissected, among which 14 (93.33; 95% confidence interval, 80.26–100.00%) had laid eggs.

### Height distribution

191 trap nights were performed to capture mosquitoes in eight towns (Table 2). Analysis of data revealed that the density of *An. minimus* decreased with an increase in area elevation, irrespective of the location of collection (human bedroom: *r* = − 0.441, *P* = 0.000; livestock building: *r* = − 0.297, *P* = 0.003). A linear model may represent the relationship between the density and height. After analysing the data of 90 densities of captured *An. minimus* and the related height values, the model in human bedrooms was *y* = 2.009 − 0.02*x* (*R* ^2^ = 0.194, *P* = 0.000), where *y* and *x* indicate the density of *An. minimus* and elevation of the area, respectively. After analysing the data of 101 densities of captured *An. minimus* and the related height values, the model in cattle shelters was *y* = 6.147 − 0.005*x* (*R* ^2^ = 0.088, *P* = 0.003).

The density of *An. sinensis* increased with an elevation < 1200 m but decreased with > 1200 m (Table 9). A quadratic model could be used to represent the relationship between the density and height. Analysis of 90 densities of captured *An. sinensis* and the related height values showed that the model in human bedrooms was *y* = − 21.017 + 0.09*x* + 0.000029*x* ^2^ (*R* ^2^ = 0.199, *P* = 0.000), where *y* and *x* indicate the density of *An. sinensis* and height, respectively. Analysing 101 light-trap-captured densities of *An. sinensis* and the related height values also revealed the following model in cattle shelters: *y* = − 77.444 + 0.373*x* + 0.000177*x* ^2^ (*R* ^2^ = 0.100, *P* = 0.006).

**Table 9:** Density of *An. minimus* and *An. sinensis* at different elevations

	Elevation (m)	Number of light-traps	Number of *An. minimus*	Density of *An. minimus* (per light-trap per night)	Number of *An. sinensis*	Density of *An. sinensis* (per light-trap per night)
Human bedroom	0 ~	46	76	1.65	16	0.35
	600 ~	40	1	0.03	1387	34.68
	1200 ~	3	0	0.00	293	97.67
	1800 ~	1	0	0.00	0	0.00
	Total	90	77	0.86	1696	18.84
Cattle shelter	0 ~	46	252	5.48	89	1.93
	600 ~	41	24	0.59	5223	127.39
	1200 ~	12	5	0.42	570	47.50
	1800 ~	2	0	0.00	0	0.00
	Total	101	281	2.78	5882	58.24

## Discussion

Although some studies have focused on community structure and receptivity to malaria in the China–Myanmar border (especially in the low-elevation areas) [6, 18, 21, 22], this study focused on other aspects to better understand *Anopheles* ecological features. Firstly, this study investigated the *Anopheles* distribution and found different community structure models at different elevation levels. The relationship between *An. minimus* density and elevation fitted well on a linear equation with one unknown model, but that between *An. sinensis* density and elevation fitted well on a quadratic equation with one unknown model. Secondly, this study found that high density, human-biting rate, and parous rate may lead to high receptivity to malaria in the border area. Finally, the slightly acidic water suited the growth of the two vectors.

The results of this study showed that the community structure of *Anopheles* was highly complex in areas below an elevation of 600 m. In these areas, the diversity indices *D* and *H* and the evenness index *E* were the highest, and the species richness index was also high up to 9. Although *An. minimus* was the major malaria vector in these areas, it was only 51% of the total *Anopheles* mosquitoes, which made the dominance index to be lowest among the four elevation level. Additionally, the proportion of *An. sinensis* and *Anopheles culicifacies* was as high as 16%. These results were slightly different with those of Yu et al. and Wang et al. [6, 21]. They reported that the first three predominant *Anopheles* species were *An. minimus*, *An. maculatus*, and *An. culicifacies*, with *An. sinensis* only accounting for < 4% [6]. These differences might be due to the different study years, changes in main cash crops, and different types of mosquito capture sites. Until early 2016, banana totally replaced rice and become the dominant cash crop in Na Bang. Furthermore, while Yu et al. and Wang et al. captured adult mosquitoes in human bedrooms, we captured mosquitoes in both human bedrooms and cattle shelters. *Anopheles minimus* belongs to four high-transmission-potential vectors in China, the rest being *An. sinensis*, *An. lesteri*, and *An. dirus* [23, 24]. *Anopheles sinensis* is a major malaria vector in China, especially northern China [23, 24], India [44, 45], Sri Lanka [46], and Iran [47]. The results of the cross-seasonal surveillance showed that the community structure was stable during the study season in the China–Myanmar border. Therefore, choosing the specific vector-control measures was more difficult in this region than in other elevation level because different targeted control measures were based on different ecological features.

The dominant species in the *Anopheles* community was *An. sinensis* at an elevation of 600–1800 m, with a dominance index > 0.9, indicating absolute predominance of the vector in the area. The results of the cross-seasonal surveillance in Ping Yuan showed that the density of *Anopheles* mosquitoes peaked from June to September and > 94% of them were *An. sinensis*, indicating low diversity and evenness during that period. The highest similarity was observed between areas with elevations of 600–1199 and 1200–1799 m, suggesting that these two areas could be combined into one area, with the target vector to control being *An. sinensis*. Although *An. sinensis* prefers biting animals such as cattle or water buffalo over humans [48, 49], its extremely high density in the area could lead to a high probability of malaria transmission. Latest research using membrane feeding assay under laboratory conditions demonstrated that the susceptibility of *An. sinensis* to *Plasmodium* *vivax* is similar to that of *Anopheles anthropophagus* [24]. In addition, *P. vivax* is a major parasite of malaria in the China–Myanmar border [50, 51]. Therefore, specific vector-control countermeasures aimed at *An. sinensis* should be strengthened in the region in case of the re-establishment of malaria.

High elevation of > 1800 m was correlated with low species richness, diversity, and evenness in the area. *Anopheles kunmingensis* was the major *Anopheles* mosquito in the high-elevation areas (> 1800 m). One study reported *An.* *kunmingensis* as the main malaria vector based on its indoor abundance, relatively high human-biting rate, and the finding of a sporozoite-positive specimen during a peak malaria season in Tengchong County, Yunnan Province, China [52]. However, the role of mosquitoes in transmission of malaria, especially in susceptibility to *Plasmodium* and receptivity to malaria, remains uncertain. Therefore, further research on *Anopheles* mosquitos is required to determine the integrated aspects of malaria transmission.

The results of this study further revealed that the human-biting rate of *An. minimus* was remarkably high in Na Bang, with the highest rate of 15 females/person/night in August. In the same month, the human-biting rate in Ping Yuan was 21 females/person/night. In addition, *An. minimus* was more likely to attack people after midnight, while *An. sinensis* before midnight. These findings necessitate the increased use of countermeasures such as bed nets and mosquito repellents for preventing vector bites from May to September, especially in August.

The seasonal abundance of *An. minimus* was significantly higher than that reported by Wang et al. [6] in Na Bang in 2012–2013. The densities in cattle shelters were higher than those in human bedrooms. *Anopheles minimus* preferred areas at low elevation and tropical areas and showed a high density in cattle shelters in June, while *An. sinensis* preferred a medium elevation and showed a high density in August. In areas of low elevation, the conditions in June and September were more suitable to the vector, although the density in these areas was < 5 females/trap/night. Moreover the seasonal peak of *An. minimus* larvae occurred in July, while that of *An. sinensis* larva occurred in August. Therefore, more mosquito-control measures such as pesticide sprays should be used in this region before June.

The parous rate of *An. sinensis* and *An. minimus* was 90.46% and 93.33%, respectively. If the duration from eclosion to laying eggs is 2.5 days, the daily survival probability of *An. sinensis* and *An. minimus* will be 96.07% and 97.28%, respectively, according to the function *p* = *M* ^1/*X*^, where *p*, *M*, and *X* refer to the daily survival probability, the parous rate, and the duration from eclosion to laying eggs, respectively. According to the MacDonald model [53], vectorial capacity (*VCAP*) [54–57], which is the indicator of receptivity to malaria, can be presented by the function $VCAP = ma \times a \times p^{n} \times 1/\left( { - \ln p} \right)$, where *a* and *n* indicate the vector biting rate and the parasite’s extrinsic incubation period that is affected by ambient temperatures, respectively. Therefore, a higher human-biting rate and ratio of vectors having laid eggs lead to a higher *VCAP*. In this study, these two parameters of *An. sinensis* and *An. minimus* in Yingjiang County showed exceedingly high values.

Due to several complicating factors, malaria in the China–Myanmar border might threaten the elimination of malaria in China [5]. Yingjiang County harbours several ethnic minorities, and their subsistence activities associated with forest areas, such as logging, banana or rubber planting, and living in planting areas during the farming season or entire year, are likely to increase the risk of infection [58]. Under conditions of high receptivity and potential exposure of the local people, if imported malaria cases occur in the county without timely and effective control, the probability of re-establishment will be extremely high. Consequently, specified vector-control countermeasures should be strengthened in these areas in case of the re-establishment of malaria, which might affect the progress of malaria elimination in China, and more public health programmes should focus on controlling malaria transmission in the China–Myanmar border region to better achieve malaria elimination in China.

### Limitation

The seasonality of species composition, density, *ma*, and parous rate was only investigated in two towns. Thus, more surveys are necessary across the four elevation levels to investigate the integrated aspects of receptivity to malaria in the China–Myanmar border.

## Conclusions

This study showed that the community structure of *Anopheles* was complex and stable during the entire epidemic season at low elevation areas in the China–Myanmar border in Yingjiang County, China. The highest similarities in vector features were observed in areas with elevations of 600–1199 and 1200–1799 m. These areas of medium elevation showed significant seasonality in the community structure (such as density, diversity, dominance, and richness). Meanwhile, the community structure was relatively simple in areas of elevations > 1800 m compared with other areas. Based on the high human-biting rate, adult and larval density, and parous rate of the two vectors, receptivity to malaria was exceedingly high in the China–Myanmar border in Yingjiang County. These findings can provide insights into the epidemiology of malaria as well as direct and quantified evidence to draw up vector control strategies and promote progress of malaria elimination in China.

## Abbreviations

WHO: World Health Organization
GMS: Greater Mekong Subregion
CDC: Centers for Disease Control and Prevention
PCR: polymerase chain reaction
HLC: human-landing catch
HDN: human-baited double-net
VCAP: vectorial capacity
